# Revascularization Treatment of Emergency Patients with Acute ST-Segment Elevation Myocardial Infarction in Switzerland: Results from a Nationwide, Cross-Sectional Study in Switzerland for 2010-2011

**DOI:** 10.1371/journal.pone.0153326

**Published:** 2016-04-14

**Authors:** Claudia Berlin, Peter Jüni, Olga Endrich, Marcel Zwahlen

**Affiliations:** 1 Institute of Social and Preventive Medicine (ISPM), University of Bern, Bern, Switzerland; 2 Applied Health Research Centre (AHRC), Li Ka Shing Knowledge Institute of St. Michael’s Hospital, and Department of Medicine, University of Toronto, Toronto, Canada; 3 Institute of Primary Health Care (BIHAM), University of Bern, Bern, Switzerland; 4 Inselspital, Bern University Hospital, Bern, Switzerland; University Hospital Medical Centre, GERMANY

## Abstract

**Background:**

Cardiovascular diseases are the leading cause of death worldwide and in Switzerland. When applied, treatment guidelines for patients with acute ST-segment elevation myocardial infarction (STEMI) improve the clinical outcome and should eliminate treatment differences by sex and age for patients whose clinical situations are identical. In Switzerland, the rate at which STEMI patients receive revascularization may vary by patient and hospital characteristics.

**Aims:**

To examine all hospitalizations in Switzerland from 2010–2011 to determine if patient or hospital characteristics affected the rate of revascularization (receiving either a percutaneous coronary intervention or a coronary artery bypass grafting) in acute STEMI patients.

**Data and Methods:**

We used national data sets on hospital stays, and on hospital infrastructure and operating characteristics, for the years 2010 and 2011, to identify all emergency patients admitted with the main diagnosis of acute STEMI. We then calculated the proportion of patients who were treated with revascularization. We used multivariable multilevel Poisson regression to determine if receipt of revascularization varied by patient and hospital characteristics.

**Results:**

Of the 9,696 cases we identified, 71.6% received revascularization. Patients were less likely to receive revascularization if they were female, and 80 years or older. In the multivariable multilevel Poisson regression analysis, there was a trend for small-volume hospitals performing fewer revascularizations but this was not statistically significant while being female (Relative Proportion = 0.91, 95% CI: 0.86 to 0.97) and being older than 80 years was still associated with less frequent revascularization.

**Conclusion:**

Female and older patients were less likely to receive revascularization. Further research needs to clarify whether this reflects differential application of treatment guidelines or limitations in this kind of routine data.

## Introduction

Mortality for cardiovascular diseases (CVDs) has continually decreased in developed countries, but CVDs are still the leading cause of death in Switzerland for both sexes [1]. More rapid and improved treatment of acute myocardial infarction (AMI) has substantially reduced CVD mortality [2]. International guidelines recommend, for optimal treatment, that AMI patients receive evidence-based therapies [3–5]. Treatment guidelines differ by type of AMI: ST-elevation myocardial infarction (STEMI) is differentiated from non-ST-elevation myocardial infarction (NSTEMI). If treatment guidelines are consistently followed, they should reduce or eliminate sex- or age-based treatment differences for patients whose clinical situations are identical.

In Switzerland, however, there is evidence that AMI patients are treated inconsistently, and that guidelines are more likely to be followed for men than for women. Radovanovic et al analyzed patients of the Swiss AMI registry (AMIS) between 1997–2011 and saw that percutaneous coronary interventions (PCI) had increased in STEMI patients overall. But, since 2006, they found that over 80% of male patients received PCI, while only 70% of women were treated with PCI [6]. Women were less likely to receive primary reperfusion (thrombolysis and PCI) and medications according to evidence-based guidelines [6,7]. Other studies documented that AMI treatment in Switzerland varied by region, hospital characteristics (presence or absence of 24 hour/7day cardiac catheterization facility), age, and number of comorbidities [7–9].

We used nation-wide hospital data of patients admitted to all Swiss hospitals in 2010 and 2011, with the goal of including all patients with acute STEMI as their main diagnosis because well-established treatment guidelines exist for these patients [3]. Our goal was to investigate the effect of patient and hospital characteristics on receipt of revascularization in acute STEMI patients accounting for hospital transfers and use of treatment information over the whole course of treatment.

## Data and Methods

### Ethics

Ethical approval was not required for this analysis of data, which are available to research institutions according to the ordinance on federal statistical monitoring activities and surveys.

### Data sets

We used two national data sets from the Swiss Federal Statistical Office (SFSO) that provided information about inpatient care and hospital infrastructure in 2010 and 2011. The first data set, *Medizinische Statistik der Krankenhäuser*, focuses on hospital stays (HS) and includes mandatory information on all patients hospitalized for at least a day, recording age, sex, place of residence, date of admission and discharge (month) of the patient, as well as main and concomitant diagnoses, and treatment provided. The second data set, *Krankenhausstatistik*, focuses on hospital characteristics (HC) and contains information on hospital infrastructure for all hospitals in Switzerland, including type of hospital, number of beds, number of physicians and nurses, number of angiography devices, CT or MRI machines, presence of an emergency room. The information in these data sets can be cross-referenced by hospital-ID. To ensure data is protected, the patient residence and the geographical location of the hospital are aggregated into zip code areas consolidated into 705 medical statistics (Medstat) regions.

Ethical approval was not required for this analysis of data, which are available to research institutions according to the ordinance on federal statistical monitoring activities and surveys.

We used the community classification data set (*Raumgliederung*) from the SFSO (reference date December 31^st^, 2010) to determine the level of urbanization of Medstat regions. The variable urban/rural region in the community classification data set included four categories: (1) main city of an agglomeration; (2) other agglomeration community; (3) isolated city; and, (4) rural community. One Medstat region usually contains more than one community. If at least one community in the Medstat region was classified as 1, 2 or 3, we coded the whole Medstat region as urban. Otherwise we coded it as rural.

### Construction of course of treatment

**Fig 1:**
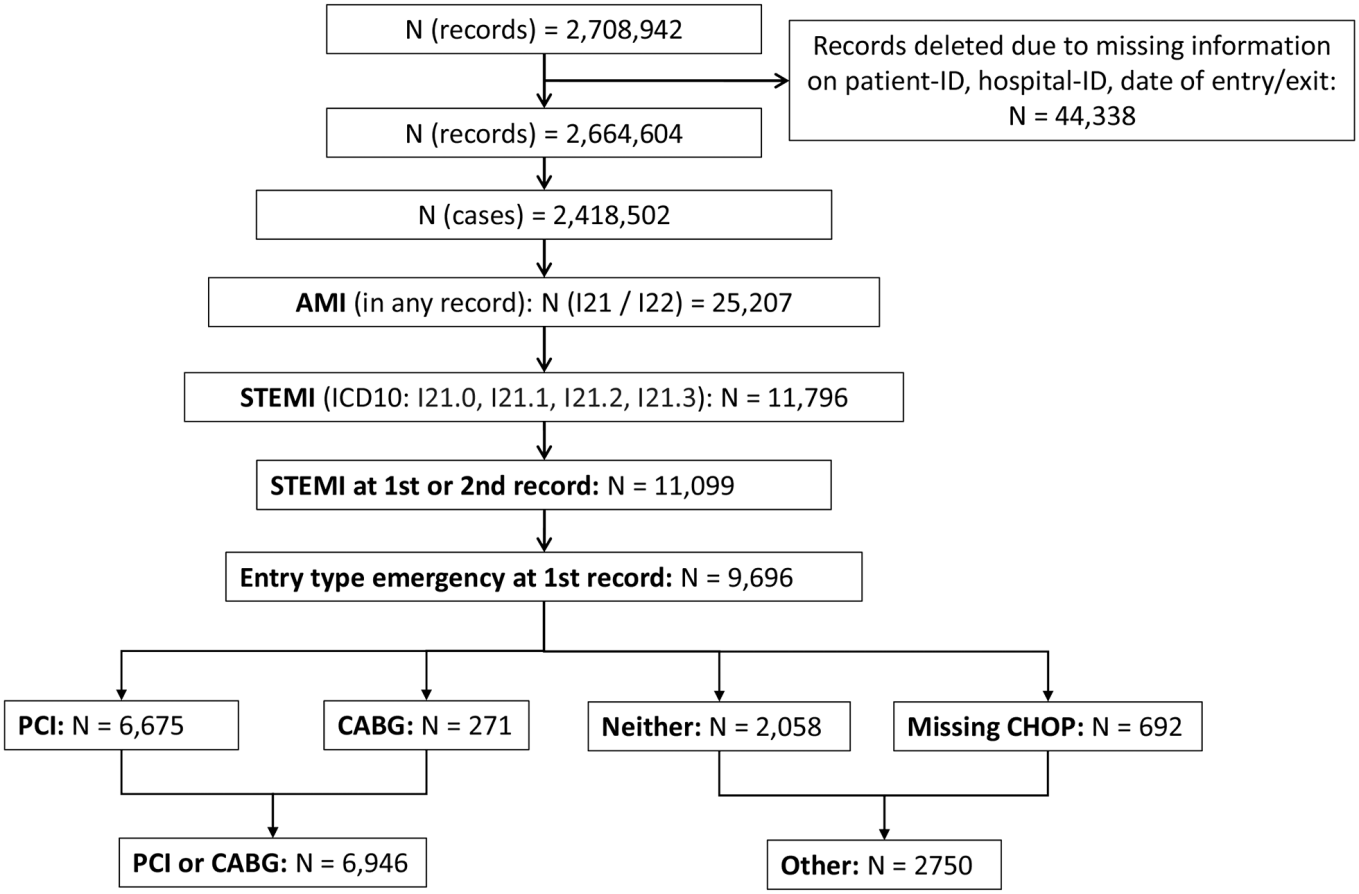
Flow chart of the selection process.

The years 2010–2011 included a total of 2,708,942 hospital stays (2010: 1.345.245; 2011: 1,363,697; Fig 1). We excluded 44,338 records for which date of entry, date of exit, hospital-ID or patient-ID were missing. Of that, the majority (99.7%) had no date of exit because these patients were hospitalized over New Year. So they had an incomplete record the year they entered the hospital and a complete record the year they were discharged. So we excluded them to eliminate duplicates and just use complete records. To protect patient anonymity, the treating hospital uses a Hash code (derived from the patient’s name, gender and date of birth) to create a patient ID, which can be used to track patients with multiple hospital stays, either in the same, or in another hospital.

Patients with several hospital stays recorded close together may have been referred between hospitals to treat the same condition. For example, STEMI patients would be recorded twice in the HS data if they were treated in one hospital for a day, and then transferred to spend several days in another hospital for further treatment. To avoid double counting of patients, we reconstructed each of these short courses of treatment, searching for sequential hospitalizations in the HS record by patient-ID, sequence number, days to next hospitalization (< = 1), and month of entry. A case may thus contain several hospital stays. For the years 2010 and 2011, we constructed 2,418,502 full cases.

### Selection of STEMI patients

The International Classification of Diseases, Injuries and Causes of Death (ICD) 10 codes were used to code main and secondary diagnoses in the HS data set. The ICD 10 code that starts with I21 is for acute transmural myocardial infarctions. To select AMI patients with STEMI specifically, we excluded cases with acute subendocardial myocardial infarction (I21.4) and unspecified acute myocardial infarction (I21.9). Cases with acute transmural myocardial infarction of anterior wall (I21.0), acute transmural myocardial infarction of inferior wall (I21.1), acute transmural myocardial infarction of other sites (I21.2) or acute transmural myocardial infarction of unspecified site (I21.3) were included. These four main diagnoses (I21.0, I21.1, I21.2, and I21.3) had to appear at the first or second hospitalization record. If mentioned on the second record we restricted the selection to cases matching predefined main diagnoses at the first record (see S1 Table). We chose these predefined diagnoses as they have similar symptoms like AMI and we wanted to identify just patients being hospitalized as an emergency with AMI. We only included cases if the first record was an emergency admission. This way we excluded patients with planned hospital stays, who were unlikely to have an acute STEMI. After applying these restrictions, we included 9,696 full treatment cases in our analysis.

### Information on treatment received

To describe the treatments that STEMI patients received, we considered all records of treatment for each case. We coded information on treatment procedures in the HS data according to the Swiss operation classification system (CHOP), which was derived and modified from the American International Classification of Diseases, Ninth Revision, Clinical Modification: ICD-9-CM, volume 3, 1994. We also coded it according to classification in All Patient Diagnosis Related Groups (APDRG). We classified the 9,696 AMI cases into four groups, based on available treatment information: (1) codes that indicated a PCI; (2) codes that indicated a coronary artery bypass grafting (CABG); (3) codes that indicated treatments other than PCI or CABG; and, (4) patients with no available treatment codes (see S2 and S3 Tables for the exact CHOP and APDRG codes we used). We then created a variable that described if patients were treated with revascularization (PCI, CABG, or both) or not.

### Patient characteristics

We assessed the following socio-demographic variables: sex, age (grouped 18–44, 45–49, 50–54, 55–59, 60–64, 65–69, 70–74, 75–79, 80–84, 85+ years), and nationality (Swiss, foreign). As a proxy for the patient’s socio-economic position, we used the patients insurance status (public, semi-private, or private), which indicates the type of insurance used for billing. Public insurance is mandatory, semi-private and private insurance are optional and paid for by patients in addition to paying public insurance. Semi private insurance is associated with a hospital stay in a two-bed room, private insurance with a stay in a single-bed room. We also used information on who referred the patient to the hospital (the patient or relatives, rescue service or a physician). To characterize the comorbidities of the patient, we used the maximum number of secondary diagnoses recorded either in the first or, if the patient was referred, the second record in the HS data (grouped into no, 1 to 2, 3 to 4, 5 to 6, and 7 or more secondary diagnoses).

### Hospital characteristics

We used the following hospital level information: presence of an angiography device; full-time equivalent (FTE) of the physicians by 1,000 cases (grouped into tertiles); type of hospital region (urban or rural); language region of the hospital (German, French or Italian); and the number of hospital stays per year. As has been done previously [10], we divided hospitals into three groups: small-volume (<15,001 cases/year), medium-volume (15,001–30,000 cases/year) or high-volume (> 30,000 cases/year) volume.

Except for treatment information and comorbidities, we derived both patient and hospital characteristics from the first hospital stay of the short course of treatment. Coding guidelines for HS data imply that the referring hospital coded the treatment when it referred a patient to another hospital for ambulatory treatment, e.g., an ambulatory PCI. This explains why we focused on the first hospital, which also took the lead in providing treatment and made the decision to refer a patient to another hospital.

### Statistical analysis

We calculated the number and percentage of patients who received revascularization and computed crude relative proportions with 95% confidence intervals (95% CI) when comparing groups of patients. We used Pearson Chi-square-test with a two-sided significance level p<0.05 to test for differences by patient or hospital characteristics. We then estimated relative proportions using multivariable multilevel Poisson regression to adjust for confounders and to identify characteristics independently associated with receiving revascularization [11]. We included patient characteristics (sex, age, nationality, bed category, maximum number of secondary diagnoses at first or second hospital, entry decision) and hospital characteristics (angiography device, fulltime equivalent of physicians per 1,000 cases, type of hospital region, language region, and hospital groups). We used multilevel Poisson regression as implemented in Stata, with the **xtpoisson** command, to account for the multilevel structure of the data (clustering of patients within the first contact hospital), and used Wald tests to examine the significance of association. In the analyses of variables with ordered levels we used the lowest level as the reference group and for dichotomous variables the level without the corresponding characteristic. We performed a sensitivity analysis by analyzing treatment with PCI alone, excluding CABG and found no major differences between the model with PCI alone and the model with PCI/CABG, hence we used PCI/CABG in all further analyses. All analyses were performed using Stata version 13 (StataCorp, College Station, TX, USA).

## Results

In Switzerland, in 2010 and 2011, 300 hospitals provided inpatient care, including specialized services for acute care, psychiatry, rehabilitation/geriatrics, or delivery/obstetrics. More than half these hospitals had an acute care division (2010: 179, 2011: 180). Of these 300 hospitals, 98 were the site of first contact in 2010, and 96 in 2011, for the 9,696 STEMI cases we included. In both years, 77 of the hospitals were located in an urban area. Of the first contact hospitals, 54 in 2010, and 56 in 2011, had at least one angiography device. Over both years, 35.4% of the included STEMI patients were initially treated in a small-volume hospital, 31.9% in a medium-volume hospital, and 32.6% in a high-volume hospital.

### Study population

The 9,696 STEMI cases (total: 9,598 different persons) were, on average, 65.8 years old (range: 18–102 years); 70.8% were male, and the mean length of stay was 11.7 days. In the group treated with revascularization (6,946 of the 9,696, or 71.6% of all), mean age was 63.3 years, 75.6% were men, and average length of stay was 11.4 days. The 2,750 patients who did not receive revascularization were, on average, 72 years old; 58.7% were male, and mean length of stay was 12.5 days.

The proportion of cases who received revascularization was highest in the 50–54 age group (83.5%), and lowest in the 85+ years age group (29.6%). The proportion was highest in the private (76.8%), and lowest in the public insurance category (70.9%). It was highest in the high-volume hospital group (81.3%), and lowest in the small-volume hospital group (55.3%). In the Italian hospital language region, 80.6% of all hospitalized STEMI cases received revascularization, while in the French language region, 70.9% received the treatment (see Table 1).

**Table 1:** Socio-demographic, socioeconomic and healthcare-related factors of the study population and crude rates of receiving a revascularization, Switzerland 2010–2011.

	N (%)	PCI/CABG (%)	Unadjusted model crude relative proportions (95% CI)
**Total**	**9,696 (100.00%)**	**6,946 (71.64%)**	
**Sex**		**p<0.0000**	**p<0.0000**
Male	6,869 (70.84%)	5,254 (76.49%)	1.0
Female	2,827 (29.16%)	1,692 (59.85%)	0.78 (0.76,0.81)
**Age groups**		**p<0.0000**	**p<0.0000**
18 to 44 years	567 (5.85%)	454 (80.07%)	1.0
45 to 49 years	727 (7.50%)	594 (81.71%)	1.02 (0.97,1.08)
50 to 54 years	988 (10.19%)	825 (83.50%)	1.04 (0.99,1.10)
55 to 59 years	1,113 (11.48%)	888 (79.78%)	1.00 (0.95,1.05)
60 to 64 years	1,189 (12.26%)	946 (79.56%)	0.99 (0.94,1.04)
65 to 69 years	1,174 (12.11%)	952 (81.09%)	1.01 (0.96,1.06)
70 to 74 years	1,003 (10.34%)	756 (75.37%)	0.94 (0.89,0.99)
75 to 79 years	1,063 (10.96%)	727 (68.39%)	0.85 (0.81,0.91)
80 to 84 years	916 (9.45%)	521 (56.88%)	0.71 (0.66,0.76)
85+ years	956 (9.86%)	283 (29.60%)	0.37 (0.33,0.41)
**Citizenship**		**p<0.0000**	**p<0.0000**
Foreign	1,731 (17.85%)	1,331 (76.89%)	1.0
Swiss	7,965 (82.15%)	5,615 (70.50%)	0.92 (0.89,0.94)
**Entry decision**		**p<0.0386**	**p<0.0382**
Herself/Himself, relatives	2,415 (24.91%)	1,693 (70.10%)	1.0
Rescue services	3,730 (38.47%)	2,659 (71.29%)	1.02 (0.98,1.05)
Physician	3,551 (36.62%)	2,594 (73.05%)	1.04 (1.01,1.08)
**Comorbidities**		**p<0.0000**	**p<0.0000**
No	276 (2.85%)	91 (32.97%)	1.0
1–2	2,298 (23.70%)	1,757 (76.46%)	2.32 (1.96,2.75)
3–4	3,072 (31.68%)	2,384 (77.60%)	2.35 (1.99,2.79)
5–6	1,908 (19.68%)	1,415 (74.16%)	2.25 (1.90,2.67)
7+	2,142 (22.09%)	1,299 (60.64%)	1.84 (1.55,2.18)
**Insurance status**		**p<0.0030**	**p<0.0013**
Public	7,700 (79.41%)	5,462 (70.94%)	1.0
Half Private	1,401 (14.45%)	1,027 (73.30%)	1.03 (1.00,1.07)
Private	595 (6.14%)	457 (76.81%)	1.08 (1.03,1.13)
**Hospital groups**		**p<0.0000**	**p<0.0000**
Small (<15001 cases)	3,435 (35.43%)	1,900 (55.31%)	1.0
Medium (15001–30000 cases)	3,097 (31.94%)	2,475 (79.92%)	1.44 (1.40,1.50)
High (>30000 cases)	3,164 (32.63%)	2,571 (81.26%)	1.47 (1.42,1.52)
**Language region**		**p<0.0000**	**p<0.0000**
German	7,140 (73.64%)	5,085 (71.22%)	1.0
French	2,052 (21.16%)	1,455 (70.91%)	1.00 (0.96,1.03)
Italian	504 (5.20%)	406 (80.56%)	1.13 (1.08,1.18)
**FTE physicians/1000 cases**		**p<0.0000**	**p<0.0000**
1. tertile (<11.86)	3,236 (33.37%)	1,932 (59.70%)	1.0
2. tertile (11.86-<17.46)	3,300 (34.03%)	2,454 (74.36%)	1.25 (1.20,1.29)
3. tertile (17.46+)	3,151 (32.50%)	2,556 (81.12%)	1.36 (1.31,1.40)
n/a	9 (0.09%)	4 (44.44%)	
**Hospital region**		**p<0.0000**	**p<0.0000**
Rural	504 (5.20%)	230 (45.63%)	1.0
Urban	9,192 (94.80%)	6,716 (73.06%)	1.60 (1.45,1.76)
**Angiography device**		**p<0.0000**	**p<0.0000**
No	1,290 (13.30%)	666 (51.63%)	1.0
Yes	8,406 (86.70%)	6,280 (74.71%)	1.45 (1.37,1.53)

### Factors associated with receiving a revascularization

The results of the univariable Poisson regression analyses showed revascularization was less common in female patients, those older than 69 and those who were Swiss (see Table 1). Patients were more likely to receive revascularization if they were referred to the hospital by their physician, had any secondary diagnoses, were in the private insurance category, were first treated in a medium- or high-volume hospital, or were hospitalized in the Italian language region. The relative proportion of revascularization treatments increased with the number of FTE of physicians per 1,000 cases. Patients treated in an urban hospital, and in hospitals with an angiography device were more likely to receive revascularization.

**Fig 2:**
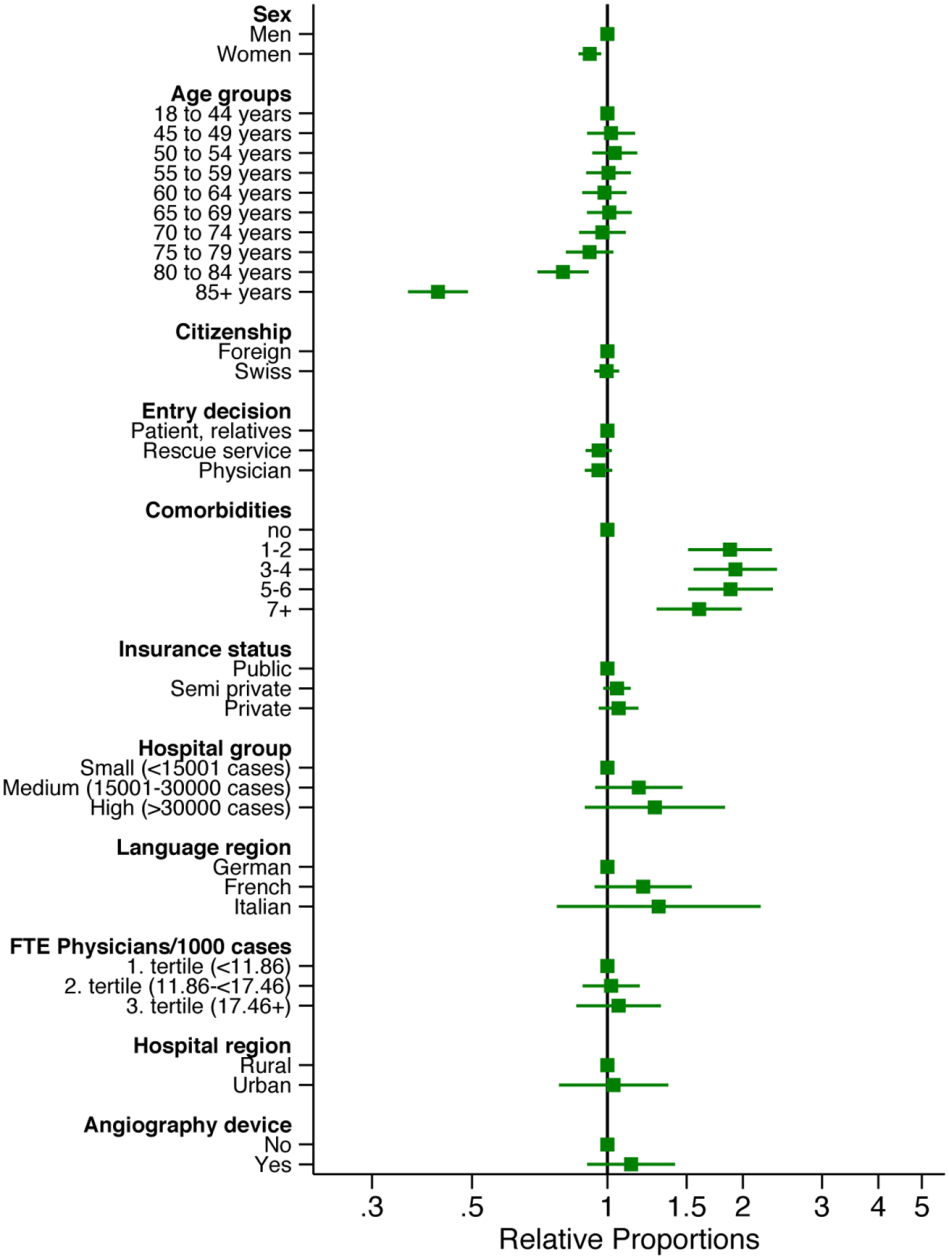
Results of multilevel Poisson regression for revascularization.

In the multivariable multilevel Poisson regression analysis most of these associations disappeared, while following associations persisted: A negative association of revascularization with being female (RP = 0.91, 95% CI: 0.86 to 0.97) and with being 80 years and older (age group 80–84 years: RP = 0.80, 95% CI: 0.70 to 0.91; age group 85+ years: RP = 0.42, 95% CI: 0.36 to 0.49). Having more comorbidities did not reduce the likelihood for revascularization (see Fig 2 and S4 Table).

**Fig 3:**
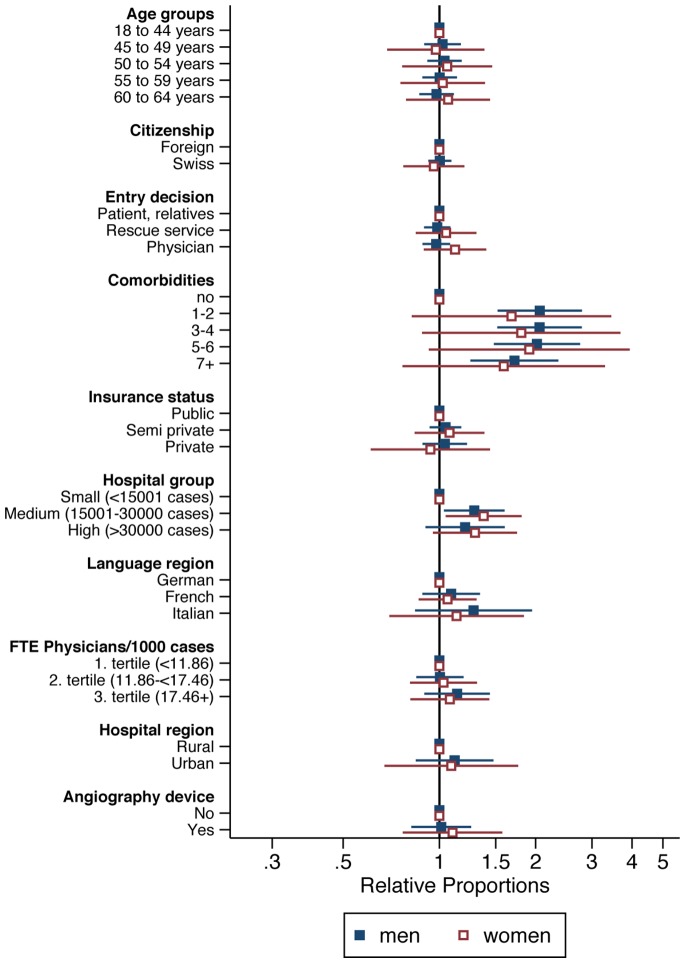
Results of multilevel Poisson regression for revascularization for male and female patients younger than 65 years.

**Fig 4:**
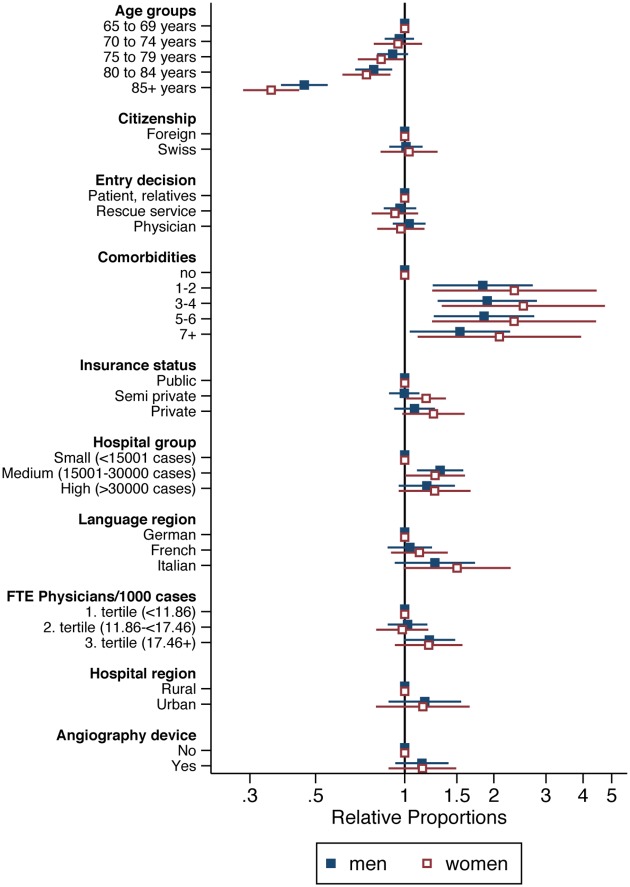
Results of multilevel Poisson regression for revascularization for male and female patients 65 years and older.

Furthermore, we present (Figs 3 and 4; S5 and S6 Tables) separate multilevel Poisson regression analyses for men and women, stratified by age (younger age group: 18–64 years, older age group: 65+ years).

Having comorbidities wasn’t a limiting factor to receive revascularization for young or old men and old women. In the adjusted analyses, revascularization was lower for men and women over 80. Women in the older age group had more often revascularization (RP = 1.18, 95% CI: 1.01 to 1.38) if their insurance status was semi-private. Young and old men as well as young women were more often treated with revascularization when admitted first to a medium size hospital (young men: RP = 1.29, 95% CI: 1.03 to 1.60; old men: RP = 1.32, 95% CI: 1.10 to 1.58; young women: RP = 1.38, 95% CI: 1.05 to 1.81).

In a sensitivity analysis we excluded cases which died the same day of admission, were 85+ years old or were admitted to hospitals with less than 3000 cases/year. This left 8,188 cases for the analysis. The stratified models showed that being hospitalized first in a small hospital led to less revascularization than an admission to a medium or high-volume hospital. The full model showed no significant association.

## Discussion

### Main findings

In patients with acute ST-segment elevation myocardial infarction admitted as emergencies to Swiss hospitals in 2010 and 2011, close to three-quarters (71.6%) received revascularization. Women were less likely than men to be treated with PCI or CABG. Independent of other patient and hospital characteristics, patients 80 years and older underwent revascularization less frequently. In younger women, we observed no incremental association with number of comorbidities. Compared to patients with no comorbidities, having comorbidities was not a barrier to receive revascularization for old male and female patients as well as for young men. Patients admitted to a medium-sized (15001–30000 hospitalizations per year) hospital were more likely to receive a PCI or CABG. In a sensitivity analysis excluding patients died the day of admission, patients being 85 years and older or admitted to hospitals with less than 3000 cases/year we found a positive trend for revascularization when admitted to a medium or high-volume hospital compared to cases first hospitalized in a small hospital.

### Limitations and strengths

Our study had some limitations. We used secondary data that was not collected for our study purpose, so we did not know why patients received no revascularization, or what kind of medication they received. For patients treated in more than one hospital, coding guidelines limit our ability to clearly determine which hospital provided which treatment [12]. We addressed this in two ways. First, for our analysis, we used the characteristics of the first hospital a patient went to. Second, we used treatment information across all hospitals in which a patient stayed during the course of treatment.

We chose the ICD-10 codes I21.0, I21.1, I21.2 and I21.3 to identify STEMI cases after consulting cardiologists and a coding expert from the Bern University hospital. ICD-10 codes do not distinguish between STEMI or NSTEMI cases [13,14]. Although they found difficulties in identifying the correct proportion of STEMI and NSTEMI on the bases of ICD-10 Alexandrescu et al. suggest to use I22.0, I22.1 and I22.8 additional to our chosen codes (I21.0-I21.3) to identify STEMI cases. When we included these ICD-10 codes, additional 44 cases were identified with almost identical results when included in the regression analyses.

We lacked specific information about the capability of a hospital to offer revascularization, and used the presence of an angiography device as a proxy without information about service hours of catheter labs (e.g. 24/7). Since treatments are coded, and diagnoses are made at the hospital where the patient is treated, coding practices may differ between hospitals e.g. due to variation in adherence to coding guidelines; this could introduce differential misclassification. The official coding guidelines are changed almost yearly. To limit the impact of changes, we restricted our analysis to 2010 and 2011. Although we used multilevel multivariable regression analyses, we cannot exclude residual confounding, bias due to variation of data collection procedures across hospitals, especially in information on prognostic factors of patients.

We examined the referral pattern per hospital group and the length of stay to consider difference in comorbidity coding. Of the 3,771 cases being transferred, 773 had no comorbidities coded in the first hospital. Considering the length of stay of these cases 88.4% (683) left the first hospital to another hospital the same day they were admitted. Therefore we believe that due to the short length of stay the registration of the comorbidities of the patient was poor in the first hospital. To overcome these differences in comorbidity coding we decided to use the maximum number of comorbidities at the first or second hospital. In a first analysis we used the number of comorbidities coded just by the first hospital. We found that having more than six comorbidities leads to less revascularization. We are convinced that the new variable of the maximum number of comorbidities is a better proxy for the real number of comorbidities of the patients. Nevertheless, this improvement is just referring to cases which were transferred.

Our study has several strengths. We analyzed standardized data of all hospitalized patients in all hospitals in Switzerland, so our results are valid for the whole country. The data set not only included patient characteristics and treatment provided, but also, via linkage to hospital characteristics data of the SFSO, it contained information on the personnel and technical infrastructure of Swiss hospitals. By constructing the course of treatment of emergency STEMI patients, we accounted for referrals and used the treatment information from all hospitals the patient visited during treatment. To our knowledge, previous Swiss studies did not take into account the treatments the patients received at all hospitals in case of hospital transfers [6–9,14]. The studies reporting on patients monitored in the AMIS registry restricted their analysis to patients exclusively treated at hospitals collaborating in the AMIS registry [6,7,9]. As 39% of all cases in our study were transferred at least once, it was important to consider all treatment information available over the whole course of treatment.

### Comparison with other studies

Like other studies of AMI patients, the receipt of optimal revascularization varied by age and gender when we adjusted for age and other patient and hospital characteristics [6,15–18]. Studies in the US and UK observed higher rates of invasive cardiac procedures in hospitals with on-site revascularization facilities [19,20]. In our univariable analysis, patients in hospitals with an angiography device more often received revascularization, but in the multivariable analysis, this association was no longer statistically significant.

Tung, et al, showed more PCI use among physicians who had high overall case volume [21]. Although the total number of annual hospitalizations does not directly reflect the frequency with which a hospital performs PCI and CABG we found a trend for less revascularization when admitted first to a small-volume hospital but the association was not significant. Just in the stratified (by age and gender) sensitivity analysis (exclusion of cases admitted to hospitals with less than 3000 cases/year, 85 years and more of age or patients which died the day of admission) this trend was significant.

Insam and colleagues analyzed hospitalizations for AMI in Switzerland before 2009 to determine if clinical management of AMI varied across seven major regions. They observed significant geographical differences and explained that Swiss hospitals are largely autonomous in defining their standards of care [8,14], but did not account for urbanization. Like studies from Canada [22,23], we found no regional differences, either between the language regions or between urban and rural hospitals.

## Conclusions

Older patients and women were less likely to receive revascularization. Further research needs to clarify whether this reflects differential application of treatment guidelines or limitations in this kind of routine data.

## Supporting Information

S1 Table Predefined main diagnosis at first hospitalization record if second hospitalization record is STEMI. (DOCX)

S2 Table CHOP and APDRG codes used to identify PCI treatment. (DOCX)

S3 Table CHOP and APDRG codes used to identify CABG treatment. (DOCX)

S4 Table Results of the multilevel Poisson regression for revascularization. (DOCX)

S5 Table Results of the multilevel Poisson regression for revascularization for male and female patients younger than 65 years. (DOCX)

S6 Table Results of the multilevel Poisson regression for revascularization for male and female patients 65 years and older. (DOCX)
