# Supplementary material for: Revascularization Treatment of Emergency Patients with Acute ST-Segment Elevation Myocardial Infarction in Switzerland: Results from a Nationwide, Cross-Sectional Study in Switzerland for 2010-2011
Source: PLoS One. 2016 Apr 14;11(4):e0153326. doi: 10.1371/journal.pone.0153326 (PMC4831744; doi:10.1371/journal.pone.0153326)
Supplement: S1 Table — (DOCX) [file pone.0153326.s001.docx]

| **ICD 10 code** | **Diagnosis** |
| --- | --- |
| I20.0 | Unstable angina |
| I20.1 | Angina pectoris with documented spasm |
| I20.8 | Other forms of angina pectoris |
| I20.9 | Angina pectoris, unspecified |
| I24.8 | Other forms of acute ischaemic heart disease |
| I24.9 | Acute ischaemic heart disease, unspecified |
| I25.1 | Atherosclerotic heart disease |
| I46.0 | Cardiac arrest with successful resuscitation |
| I49.0 | Other cardiac arrhythmias |
| R06.0 | Dyspnoea |
| R07.1 | Chest pain on breathing |
| R07.2 | Precordial pain |
| R07.3 | Other chest pain |
| R07.4 | Chest pain, unspecified |
| R57.0 | Cardiogenic shock |
| R57.9 | Shock, unspecified |
