# Supplementary material for: Revascularization Treatment of Emergency Patients with Acute ST-Segment Elevation Myocardial Infarction in Switzerland: Results from a Nationwide, Cross-Sectional Study in Switzerland for 2010-2011
Source: PLoS One. 2016 Apr 14;11(4):e0153326. doi: 10.1371/journal.pone.0153326 (PMC4831744; doi:10.1371/journal.pone.0153326)
Supplement: S2 Table — (DOCX) [file pone.0153326.s002.docx]

| **CHOP code indicating PCI** | **Explanation** |
| --- | --- |
| Z00.45 | One stent inserted |
| Z00.46 | Two stents inserted |
| Z00.47 | Three stents inserted |
| Z00.48 | Four or more stents inserted |
| Z00.66 | Percutaneous transluminal coronary angioplasty [PTCA] or coronary atherectomy |
| Z36.0 | Removal of coronary artery obstruction and insertion of stent(s) |
| Z36.06 | Insertion of non-drug-eluting coronary artery stent(s) |
| Z36.07 | Insertion of drug-eluting coronary artery stent(s) |
| **APDRG code indicating PCI** | **Explanation** |
| 112 | Percutaneous cardiovascular intervention without acute myocardial infarction, cardiac insufficiency/heart failure or shock |
| 1112 | Percutaneous cardiovascular intervention without acute myocardial infarction, cardiac insufficiency/heart failure or shock, with multiple interventions |
| 808 | Percutaneous cardiovascular intervention with acute myocardial infarction, cardiac insufficiency/heart failure or shock |
| 1808 | Percutaneous cardiovascular intervention with acute myocardial infarction, cardiac insufficiency/heart failure or shock, with multiple interventions |
