# Supplementary material for: Revascularization Treatment of Emergency Patients with Acute ST-Segment Elevation Myocardial Infarction in Switzerland: Results from a Nationwide, Cross-Sectional Study in Switzerland for 2010-2011
Source: PLoS One. 2016 Apr 14;11(4):e0153326. doi: 10.1371/journal.pone.0153326 (PMC4831744; doi:10.1371/journal.pone.0153326)
Supplement: S3 Table — (DOCX) [file pone.0153326.s003.docx]

| **CHOP code indicating CABG** | **Explanation** |
| --- | --- |
| Z36.1 | Bypass anastomosis for heart revascularization |
| Z36.10 | Aortocoronary bypass for heart revascularization, not otherwise specified |
| Z36.11 | (Aorto)coronary bypass of one coronary artery |
| Z36.12 | (Aorto)coronary bypass of two coronary arteries |
| Z36.13 | (Aorto)coronary bypass of three coronary arteries |
| Z36.14 | (Aorto)coronary bypass of four or more coronary arteries |
| Z36.15 | Single internal mammary-coronary artery bypass |
| Z36.16 | Double internal mammary-coronary artery bypass |
| Z36.19 | Other bypass anastomosis for heart revascularization |
| **APDRG code indicating CABG** | **Explanation** |
| 106 | Coronary bypass, with intracardiac catheter |
| 107 | Coronary bypass, without intracardiac catheter |
| 546 | Coronary bypass, with severe complications |
