# Supplementary material for: Revascularization Treatment of Emergency Patients with Acute ST-Segment Elevation Myocardial Infarction in Switzerland: Results from a Nationwide, Cross-Sectional Study in Switzerland for 2010-2011
Source: PLoS One. 2016 Apr 14;11(4):e0153326. doi: 10.1371/journal.pone.0153326 (PMC4831744; doi:10.1371/journal.pone.0153326)
Supplement: S4 Table — (DOCX) [file pone.0153326.s004.docx]

|  | **All patients** |
| --- | --- |
|  | **(95% CI)** |
| **Sex** | **p<0.0023** |
| Male | 1.0 |
| Female | 0.91 (0.86,0.97) |
| **Age groups** | **p<0.0000** |
| 18 to 44 years | 1.0 |
| 45 to 49 years | 1.02 (0.90,1.15) |
| 50 to 54 years | 1.04 (0.93,1.16) |
| 55 to 59 years | 1.01 (0.90,1.13) |
| 60 to 64 years | 0.99 (0.88,1.10) |
| 65 to 69 years | 1.01 (0.90,1.13) |
| 70 to 74 years | 0.97 (0.86,1.10) |
| 75 to 79 years | 0.91 (0.81,1.03) |
| 80 to 84 years | 0.80 (0.70,0.91) |
| 85+ years | 0.42 (0.36,0.49) |
| **Citizenship** | **p<0.9026** |
| Foreign | 1.0 |
| Swiss | 1.00 (0.93,1.06) |
| **Entry decision** | **p<0.3483** |
| Herself/Himself, relatives | 1.0 |
| Rescue services | 0.96 (0.89,1.02) |
| Physician | 0.96 (0.89,1.02) |
| **Comorbidities** | **p<0.0000** |
| No | 1.0 |
| 1 - 2 | 1.87 (1.51,2.32) |
| 3 - 4 | 1.92 (1.56,2.38) |
| 5 - 6 | 1.88 (1.51,2.33) |
| 7+ | 1.60 (1.29,1.99) |
| **Insurance status** | **p<0.2585** |
| Public | 1.0 |
| Half Private | 1.05 (0.98,1.13) |
| Private | 1.06 (0.96,1.17) |
| **Hospital groups** | **p<0.2157** |
| Small (<15001 cases) | 1.0 |
| Medium (15001-30000 cases) | 1.17 (0.94,1.47) |
| High (>30000 cases) | 1.27 (0.89,1.82) |
| **Language region** | **p<0.2404** |
| German | 1.0 |
| French | 1.20 (0.94,1.54) |
| Italian | 1.30 (0.77,2.19) |
| **FTE physicians/1000 cases** | **p<0.8691** |
| 1. tertile (<11.86) | 1.0 |
| 2. tertile (11.86-<17.46) | 1.02 (0.88,1.18) |
| 3. tertile (17.46+) | 1.06 (0.85,1.31) |
| **Hospital region** | **p<0.8227** |
| Rural | 1.0 |
| Urban | 1.03 (0.78,1.37) |
| **Angiography device** | **p<0.2886** |
| No | 1.0 |
| Yes | 1.13 (0.90,1.41) |
