# Supplementary material for: Revascularization Treatment of Emergency Patients with Acute ST-Segment Elevation Myocardial Infarction in Switzerland: Results from a Nationwide, Cross-Sectional Study in Switzerland for 2010-2011
Source: PLoS One. 2016 Apr 14;11(4):e0153326. doi: 10.1371/journal.pone.0153326 (PMC4831744; doi:10.1371/journal.pone.0153326)
Supplement: S5 Table — (DOCX) [file pone.0153326.s005.docx]

|  | **Men** | **Women** |
| --- | --- | --- |
|  | **(95% CI)** | **(95% CI)** |
| **Age groups** | **p<0.8529** | **p<0.9728** |
| 18 to 44 years | 1.0 | 1.0 |
| 45 to 49 years | 1.02 (0.90,1.17) | 0.97 (0.69,1.38) |
| 50 to 54 years | 1.04 (0.92,1.17) | 1.06 (0.77,1.46) |
| 55 to 59 years | 1.00 (0.88,1.14) | 1.02 (0.76,1.39) |
| 60 to 64 years | 0.98 (0.87,1.11) | 1.06 (0.79,1.44) |
| **Citizenship** | **p<0.9426** | **p<0.7214** |
| Foreign | 1.0 | 1.0 |
| Swiss | 1.00 (0.92,1.09) | 0.96 (0.77,1.20) |
| **Entry decision** | **p<0.9014** | **p<0.6067** |
| Herself/Himself, relatives | 1.0 | 1.0 |
| Rescue services | 0.98 (0.90,1.08) | 1.05 (0.84,1.31) |
| Physician | 0.98 (0.89,1.08) | 1.12 (0.89,1.40) |
| **Comorbidities** | **p<0.0000** | **p<0.3332** |
| No | 1.0 | 1.0 |
| 1 - 2 | 2.06 (1.52,2.79) | 1.68 (0.82,3.45) |
| 3 - 4 | 2.06 (1.52,2.79) | 1.80 (0.88,3.68) |
| 5 - 6 | 2.02 (1.48,2.75) | 1.91 (0.93,3.94) |
| 7+ | 1.72 (1.25,2.36) | 1.59 (0.77,3.29) |
| **Insurance status** | **p<0.7019** | **p<0.7939** |
| Public | 1.0 | 1.0 |
| Half Private | 1.04 (0.93,1.17) | 1.08 (0.84,1.38) |
| Private | 1.04 (0.89,1.22) | 0.94 (0.61,1.44) |
| **Hospital groups** | **p<0.0655** | **p<0.0703** |
| Small (<15001 cases) | 1.0 | 1.0 |
| Medium (15001-30000 cases) | 1.29 (1.03,1.60) | 1.38 (1.05,1.81) |
| High (>30000 cases) | 1.20 (0.91,1.60) | 1.29 (0.95,1.75) |
| **Language region** | **p<0.4001** | **p<0.7709** |
| German | 1.0 | 1.0 |
| French | 1.09 (0.88,1.34) | 1.06 (0.86,1.31) |
| Italian | 1.28 (0.84,1.95) | 1.13 (0.70,1.84) |
| **FTE physicians/1000 cases** | **p<0.5446** | **p<0.8723** |
| 1. tertile (<11.86) | 1.0 | 1.0 |
| 2. tertile (11.86-<17.46) | 1.00 (0.85,1.19) | 1.03 (0.81,1.31) |
| 3. tertile (17.46+) | 1.14 (0.90,1.44) | 1.08 (0.81,1.43) |
| **Hospital region** | **p<0.4421** | **p<0.7278** |
| Rural | 1.0 | 1.0 |
| Urban | 1.12 (0.84,1.48) | 1.09 (0.67,1.76) |
| **Angiography device** | **p<0.9030** | **p<0.6044** |
| No | 1.0 | 1.0 |
| Yes | 1.01 (0.82,1.26) | 1.10 (0.77,1.57) |
