# Supplementary material for: Revascularization Treatment of Emergency Patients with Acute ST-Segment Elevation Myocardial Infarction in Switzerland: Results from a Nationwide, Cross-Sectional Study in Switzerland for 2010-2011
Source: PLoS One. 2016 Apr 14;11(4):e0153326. doi: 10.1371/journal.pone.0153326 (PMC4831744; doi:10.1371/journal.pone.0153326)
Supplement: S6 Table — (DOCX) [file pone.0153326.s006.docx]

|  | **Men** | **Women** |
| --- | --- | --- |
|  | **(95% CI)** | **(95% CI)** |
| **Age groups** | **p<0.0000** | **p<0.0000** |
| 65 to 69 years | 1.0 | 1.0 |
| 70 to 74 years | 0.96 (0.86,1.07) | 0.95 (0.79,1.14) |
| 75 to 79 years | 0.91 (0.81,1.03) | 0.83 (0.69,1.00) |
| 80 to 84 years | 0.79 (0.68,0.91) | 0.74 (0.62,0.89) |
| 85+ years | 0.46 (0.38,0.55) | 0.35 (0.28,0.44) |
| **Citizenship** | **p<0.8817** | **p<0.7658** |
| Foreign | 1.0 | 1.0 |
| Swiss | 1.01 (0.89,1.15) | 1.03 (0.83,1.29) |
| **Entry decision** | **p<0.4203** | **p<0.6750** |
| Herself/Himself, relatives | 1.0 | 1.0 |
| Rescue services | 0.96 (0.85,1.09) | 0.93 (0.77,1.11) |
| Physician | 1.04 (0.91,1.18) | 0.97 (0.81,1.17) |
| **Comorbidities** | **p<0.0001** | **p<0.0135** |
| No | 1.0 | 1.0 |
| 1 - 2 | 1.83 (1.24,2.71) | 2.35 (1.24,4.44) |
| 3 - 4 | 1.90 (1.29,2.79) | 2.52 (1.33,4.74) |
| 5 - 6 | 1.85 (1.25,2.74) | 2.34 (1.24,4.43) |
| 7+ | 1.54 (1.04,2.27) | 2.09 (1.11,3.94) |
| **Insurance status** | **p<0.6274** | **p<0.0359** |
| Public | 1.0 | 1.0 |
| Half Private | 1.00 (0.89,1.12) | 1.18 (1.01,1.38) |
| Private | 1.08 (0.92,1.26) | 1.25 (0.98,1.59) |
| **Hospital groups** | **p<0.0102** | **p<0.0873** |
| Small (<15001 cases) | 1.0 | 1.0 |
| Medium (15001-30000 cases) | 1.32 (1.10,1.58) | 1.27 (1.00,1.60) |
| High (>30000 cases) | 1.19 (0.95,1.48) | 1.26 (0.95,1.67) |
| **Language region** | **p<0.3227** | **p<0.1167** |
| German | 1.0 | 1.0 |
| French | 1.04 (0.88,1.24) | 1.12 (0.90,1.40) |
| Italian | 1.26 (0.93,1.73) | 1.50 (0.99,2.28) |
| **FTE physicians/1000 cases** | **p<0.1582** | **p<0.2855** |
| 1. tertile (<11.86) | 1.0 | 1.0 |
| 2. tertile (11.86-<17.46) | 1.02 (0.88,1.19) | 0.98 (0.80,1.20) |
| 3. tertile (17.46+) | 1.21 (0.99,1.48) | 1.21 (0.93,1.57) |
| **Hospital region** | **p<0.2783** | **p<0.4489** |
| Rural | 1.0 | 1.0 |
| Urban | 1.17 (0.88,1.55) | 1.15 (0.80,1.66) |
| **Angiography device** | **p<0.2043** | **p<0.3051** |
| No | 1.0 | 1.0 |
| Yes | 1.14 (0.93,1.41) | 1.15 (0.88,1.49) |
